# Geographic inequalities in non-acute healthcare supply: evidence from Ireland

**DOI:** 10.12688/hrbopenres.13412.1

**Published:** 2021-10-04

**Authors:** Samantha Smith, Brendan Walsh, Maev-Ann Wren, Steve Barron, Edgar Morgenroth, James Eighan, Seán Lyons

**Affiliations:** 1 Centre for Health Policy and Management, Trinity College Dublin, Dublin 2, Ireland; 2 Economic and Social Research Institute, Sir John Rogerson's Quary, Whitaker Square, Dublin 2, Ireland; 3 Department of Economics, Trinity College Dublin, Dublin 2, Ireland; 4 University College Dublin, Dublin 4, Ireland; 5 DCU Business School, Dublin City University, Dublin 9, Ireland; 6 Indecon, Indecon House, Dublin 4, Ireland

**Keywords:** geographic inequalities, geographic inequity, non-acute care, primary & community care, historical budgeting, Ireland

## Abstract

**Background: **Recent reforms in Ireland, as outlined in Sláintecare, the report of the cross-party parliamentary committee on health, are focused on shifting from a hospital-centric system to one where non-acute care plays a more central role. However, these reforms were embarked on in the absence of timely and accurate information about the capacity of non-acute care to take on a more central role in the system. To help address this gap, this paper outlines the most comprehensive analysis to date of geographic inequalities in non-acute care supply in Ireland.

**Methods: **Data on the supply of 10 non-acute services including primary care, allied health, and care for older people, were collated. Per capita supply for each service is described for 28 counties in Ireland (Tipperary and Dublin divided into North and South), using 2014 supply and population data. To examine inequity in the geographic distribution of services, raw population in each county was adjusted for a range of needs indicators.

**Results: **The findings show considerable geographic inequalities across counties in the supply of non-acute care. Some counties had low levels of supply of several types of non-acute care. The findings remain largely unchanged after adjusting for need, suggesting that the unequal patterns of supply are also inequitable.

**Conclusions: **In the context of population changes and the influence of non-need factors, the persistence of historical budgeting in Ireland has led to considerable geographic inequities in non-acute supply, with important lessons for Ireland and for other countries. Such inequities come into sharp relief in the context of COVID-19, where non-acute supply plays a crucial role in ensuring that acute services are preserved for treating acutely ill patients.

## Introduction

Integration is at the centre of global health strategies to achieve people-centred health services ^1, 2^. One of the key goals outlined in global and European strategies to improve integration is the need to move away from hospital- and disease-based curative care models, towards services that prioritise primary and community care services and the ‘co-production’ of health ^1: p7, 3^. In Ireland, integration forms a central part of recent reforms endorsed by the cross-party parliamentary committee on health ^4^. Burke *et al.* ^5^ summarise the key aims of the “Sláintecare” strategy: to establish a universal, single-tier health service where patients are treated solely on the basis of need, and a “reorienting of the health system ‘towards integrated primary and community care consistent with the highest quality of patient safety’” ^5: p1278^. However, these reforms have been embarked on in the absence of timely and accurate information about the capacity of primary and community care (summarised as ‘non-acute care’) to take on a more central role in the system. While Sláintecare acknowledges the importance of increasing non-acute workforce capacity, up to now there has been no systematic analysis of the patterns of supply of these services across the country. To help address this gap, this paper outlines the most comprehensive analysis to date of geographic inequalities (and inequities) in non-acute care supply in Ireland.

The Irish healthcare system provides an important example of a system that was traditionally ‘hospital-centric’ ^6^, and the lower priority paid to non-acute services is evidenced by the absence of any national dataset detailing the number, location and catchment areas for non-acute services. Rather, several disparate data sources provide some information on the supply of publicly provided, and very limited information on the supply of private, non-acute services. This has made it difficult to characterise the current supply of non-acute care and to undertake the research needed to help improve health policy.

Ireland also provides an example of a healthcare system where widespread historical budgeting persists. The absence of data has hindered the development of a formal population-based resource allocation formula for non-acute care ^7^, another goal in the current reforms ^8^. Many countries have adopted population-based resource allocation models to allocate supply in line with population health need characteristics (e.g., England ^9^). There are examples of resource allocation formulae in Ireland for specific services (e.g., activity-based funding in acute care). However, historical budgeting persists in non-acute care ^8^, and resource allocations are unlikely to have kept pace with changing population needs and demographic patterns across the country, and there is anecdotal evidence of geographic inequalities in supply ^4^.

### Background: measuring geographic equity in healthcare supply

Access to healthcare is a broad concept that encompasses availability and accessibility, affordability, and acceptability of services, and the degree of fit between individuals and these different dimensions ^10– 12^. Many empirical studies on access focus on one aspect of this multifaceted concept, and there is a large body of work that interprets access as a supply concept (the focus of this paper), concentrating on geographic availability of services using provider–population ratios ^11, 13– 16^. For example, UK researchers used this approach to examine the geographic distribution of general practitioners (GPs) from the mid-1970s to the 2000s, with modifications to the supply ratios to take account of geographic variations in healthcare needs ^14– 17^. We use supply ratios to examine geographic patterns of supply of non-acute services in Ireland, drawing on the methods used in the UK to adjust for healthcare needs.

Simple comparisons of supply-to-population ratios across different geographic regions provide a good indication of patterns of geographic inequality. This is the most common way in which geography is used in assessing equality and/or equity in healthcare access ^18^, allowing international organisations (e.g., Eurostat) to undertake cross-country comparisons of health and social care supply. Supply ratios are useful for broad comparisons of supply across large areas, and are used by policymakers to set minimal standards of supply and to identify underserved areas ^19^.

Other studies have focused on what has been referred to as spatial accessibility ^19^. These studies involve models (e.g., Two-step floating catchment area, 2SFCA) that move beyond simple provider–population ratios to incorporate both healthcare availability (i.e., level of supply) and accessibility (i.e., distance/time between patient location and healthcare facility). The 2SFCA method and other related techniques have been applied to a wide range of countries and healthcare services: for example, variations in geographic accessibility of different aspects of primary care in Wales ^20^, Texas, US ^21^, Ontario, Canada ^22^, and ambulatory and inpatient services in Germany ^23^. Analysis of spatial accessibility in healthcare in Ireland has been focused on GPs and long-term residential care (LTRC) ^24– 27^ but extending this type of analysis to other non-acute healthcare services is hindered by the absence of adequately geocoded data.

This paper makes four core contributions to the literature for healthcare policymakers. First, the analysis demonstrates how the persistence of historic budgeting can lead to considerable inequities in supply of non-acute care. Second, in the absence of detailed geocoded data, simple provider-to-population ratios, with adjustments for healthcare need factors, can be used to give a comprehensive description of non-acute care in a country, and identify notable regional patterns. Third, while localised decision-making facilitates better matching of supply with local demand, the Irish experience underlines the importance of appropriate data and guidance from the national level to help address any geographic imbalances in supply. Fourth, the analysis highlights important regions requiring attention from Irish policymakers undertaking comprehensive healthcare reforms.

### Institutional context

Healthcare organisation in Ireland has undergone several changes in the past 15 years, with implications for how local non-acute care services are allocated. The nationally based Health Service Executive (HSE) replaced (in 2005) 11 former regional Health Boards that had considerable autonomy in developing local services, with local political representation on the boards. The HSE replaced an older system comprised of local services that had developed in different ways ^28^. Since 2005, the HSE has overseen numerous changes in the organisation of local non-acute services from 32 Local Health Offices (LHOs), to 17 Integrated Service Areas (never fully implemented), to nine Community Health Organisations (CHOs), comprising 96 Community Health Networks (CHNs). CHNs cover an average population of 50,000 and provide the structure for integrating primary and social care. In 2019, six Regional Health Areas (RHAs) were proposed, merging hospital groups with CHOs to ensure integration across acute and non-acute care, with each RHA having its own budget and greater autonomy at local level ^29^. We examined the 10 most central non-acute healthcare services in Ireland ^30^, representative of the key professions that make up CHNs.

The extent of healthcare affordability for patients is an important element in facilitating access to healthcare. Ireland is unusual amongst other high-income European countries because it does not offer universal access to primary care ^5^ and there is a lack of clarity around entitlement to other non-acute care ^4^. For GP care, approximately 33% of the population have a Medical Card (MC) which covers free GP visits (and cover for other primary care and acute care including prescription medicines ^31^). A further 11% have a GP Visit Card (GPVC) that covers free GP care only ^32^. MCs are granted mainly on the basis of an income-based means test but some are also granted on a discretionary basis to patients with health needs for whom paying for healthcare would cause ‘undue financial hardship’ ^31^. Since 2015, GPVCs are available for children under six years of age and adults aged 70+. The rest of the population pays out-of-pocket for GP care (average €52.50 per visit ^33^). More than 40% of the population have supplementary private health insurance which mainly covers acute care ^34^. For other non-acute care, it is acknowledged that there is ‘huge variety’ in access ‘depending on geographic location and existing supply in that area’, and in practice priority is often given to MC holders ^4: p45, 35^. This paper provides a more rigorous analysis of available data to move beyond this anecdotal understanding of variable access to non-acute care services in Ireland.

## Methods

### Geographic setting

There are 26 administrative counties in the Republic of Ireland, ranging in population size from 0.32m (Leitrim) to 1.35m (Dublin), with a total population of 4.8 million people ^36^. Ireland has a low population density, with a large dispersed rural population. Per capita non-acute supply is described for 28 counties (Tipperary and Dublin are divided into North and South) using 2014 ESRI population estimates (described in detail in previous applications ^37, 38^). This level of aggregation has the advantage that counties are stable across time (aligning well with, but independent of, potentially changing HSE administrative structures) and reflect definitions of catchment areas for non-acute services, which are designed to be community-based services serving clients in their immediate locality.

### Supply and needs data

This paper draws on several data sources on the supply of 10 mainly publicly employed non-acute services, including private supply where available:

General practitioners: GPs in Ireland are self-employed private practitioners. A large proportion hold a state General Medical Services (GMS) contract to provide GP care that is free at the point of use to MC and GPVC holders ^39, 40^. Data on GP supply are based on extracts from the Irish College of General Practitioners database and the Irish Medical Directory in September 2014 ^24, 41^. GPs in training (668) were not included due to a lack of information on location and whole-time equivalent (WTE) activity ^42^. Headcounts were converted to WTEs using recent survey data on self-reported full-time/part-time practices, disaggregated by sex, by a representative sample of GPs in Ireland ^43^.

Community nurses (CNs) and allied health professionals: the HSE employs community nurses (Public Health Nurses (PHNs) and community Registered General Nurses) to provide a wide range of non-acute services to individuals within a geographic area ^44, 45^ in health centres and in individuals’ homes. Allied health professionals including physiotherapists, occupational therapists, speech and language therapists and others (podiatrists & chiropodists, counsellors & psychologists, social workers) deliver services in health centres or individuals’ homes. These can include private as well as publicly employed professionals. Data on the WTE supply (by grade, LHO, agency) of publicly employed community nurses and allied health professionals were obtained from the Health Service Personnel Census (HSPC), December 2014 ^46^.

Private physiotherapists: data on private and state-subsidised voluntary physiotherapists were taken from the register of members of the Irish Society of Chartered Physiotherapists ( ISCP). WTEs were estimated using data from an online survey of ISCP members, as previously described ^47^.

Care for older people: home carers provide domestic support and more intensive care where needed, to older people in their own home. Approximately 75% of formal home care is publicly financed and provided by a mix of state-run, not-for-profit, and for-profit organisations ^30^. LTRC is mainly provided in private nursing homes although the majority of care is publicly financed ^30^. Data on the number of publicly financed home care hours provided under the two home care schemes running in 2014 (Home Help and Home Care Package schemes) were obtained from the HSE. The 2015 data on the supply of LTRC beds were based on combined datasets maintained by the Health Information and Quality Authority (HIQA) (responsible for regulation in long-term care), and the Department of Health (DOH).

Needs data: To adjust county populations for need, data on the population aged 65+ and 85+ were available in the 2014 ESRI population estimates; data from the Central Statistics Office ( CSO) were used to estimate mortality and disability rates by county; data on the number of people with MCs and GPVCs by LHO were provided by the Primary Care Reimbursement Service ( PCRS); and data from the PCRS and the Department of Pharmacology and Therapeutics, Trinity Centre for Health Sciences in St. James's Hospital, Dublin were used to estimate chronic illness levels amongst MC holders.

### Methods

Supply data were assigned to counties based on address (e.g., a GP surgery, a nursing home), using boundary files for counties provided by the CSO. Where the address was unavailable and the supply data were assigned to aggregate areas that cross county borders (e.g., some LHOs), the supply data (e.g., number of nurses) were redistributed from the aggregate areas to counties based on share of population. The population in each LHO disaggregated by county was received from the Health Intelligence Unit of the HSE. Data on MC and GPVC numbers by LHO were assigned to counties in the same way.

Metrics presented: supply per capita, location quotients (ratio of area supply per capita to national supply per capita ^17, 18^), and Gini coefficients were estimated. Supply was measured in terms of WTEs for personnel (GPs, community nurses, etc), beds for LTRC, and hours for home care supply.

Needs adjustment: to examine potential sources of inequity in the geographic distribution of non-acute services, the raw population in each county was adjusted for each of the following needs indicators independently: age (65+ and 85+), mortality, disability, MC status and a measure of morbidity among MC holders. The choice of indicators can be categorised using Andersen’s Behavioural Model of Health Services Use ^12^ and include predisposing characteristics (i.e., age), need (i.e., mortality, disability, chronic illness levels) and enabling factors (i.e., MC coverage). For ease of presentation these are collectively referred to as ‘needs’ indicators. The needs adjustment methods are based on analysis of GP distribution in the UK ^14– 16^. The general approach involved adjusting the population in each study area by a specific need indicator, and then re-calculating the ratios of supply per capita on the basis of the adjusted population (e.g., number of GPs per person with disability in each area). A range of needs adjustments were applied, reflecting the complexity of, and the challenges in measuring healthcare need ^48^.

## Results

### Inequality in supply

Table 1 shows national average supply per capita, together with the Gini coefficient, for each of the 10 non-acute services in Ireland in 2014, prior to adjustment for need. The Gini coefficient ranges from 0.091 for LTRC to 0.615 for publicly employed podiatrists and chiropodists, indicating substantial geographic inequality in non-acute supply, but also that the degree of inequality varies across the services.

**Table 1.** Supply of non-acute care, Ireland, 2014.

	Non-Acute Primary and Community Care (WTEs)								Care for Older People	
	GP	PHN/CN	PT ^1^	OT	SLT	P&C	CO&PSY	SW	LTRC ^2^ Beds	Home Care Hours ^2^
Ireland: national supply:										
Supply per 10,000 population	5.7	5.4	3.6	2.2	1.5	0.1	1.8	1.5	49.8	24.1
Gini coefficient	0.096	0.125	0.116	0.171	0.12	0.615	0.168	0.214	0.091	0.101

Key:        GP: General Practitioner (private practitioners)                PHN/CN: Public Health Nurse/Community Registered General Nurse (publicly employed)                PT: Physiotherapist (publicly employed and private practitioners)                OT: Occupational Therapist (publicly employed)                SLT: Speech & Language Therapist (publicly employed)                P&C: Podiatrists & Chiropodists (publicly employed)                CO&PSY: Counsellors & Psychologists (publicly employed)                SW: Social Worker (publicly employed)                LTRC: Long-term Residential Care (public and private) Sources:  Secondary data collated by the authors, see Data & Methods Notes:     1. Physiotherapy supply includes public and private supply               2. Supply of long-term residential care beds per 1,000 population aged 65+; supply of home care hours per population aged 65+

**Figure 1.**
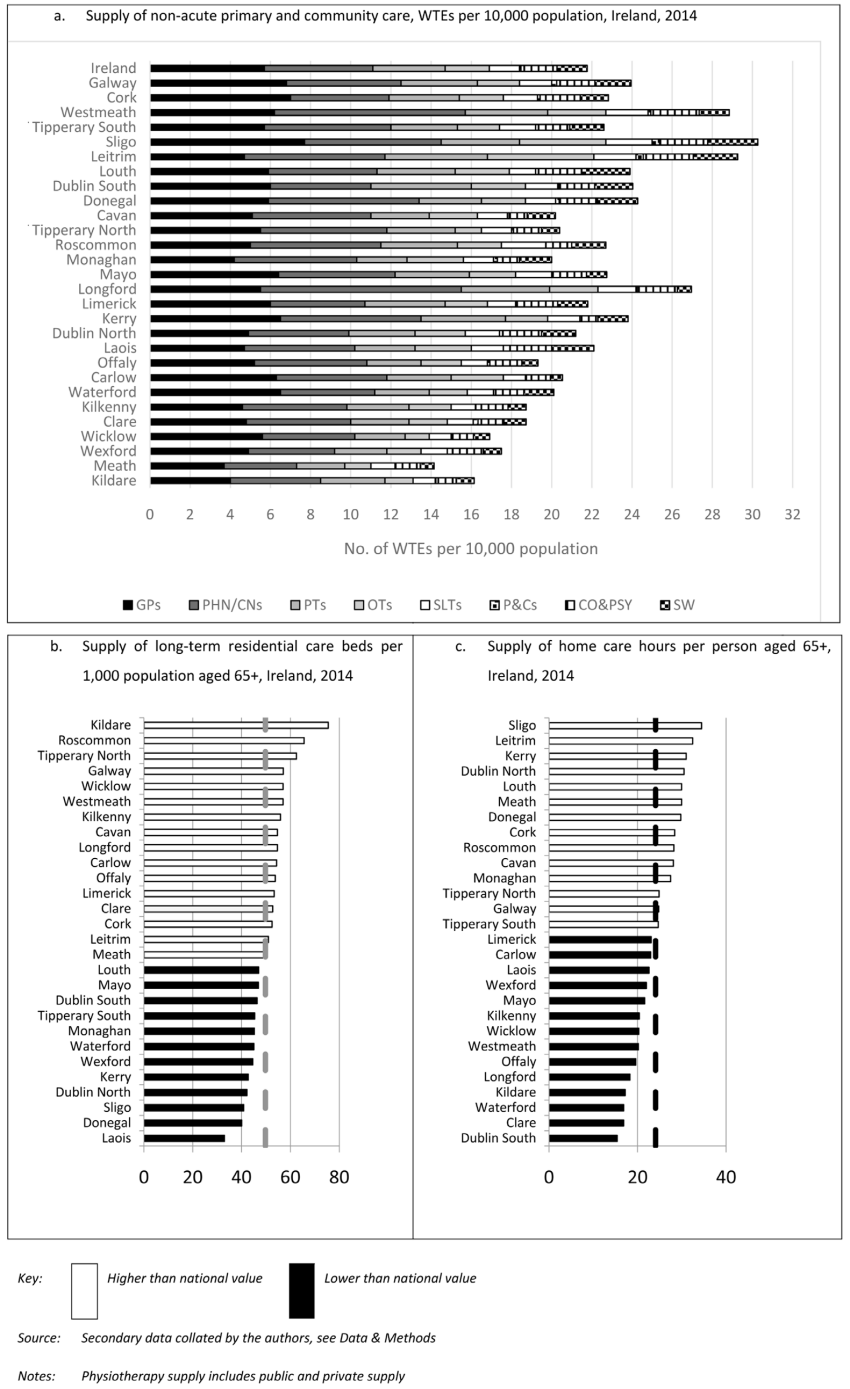
Estimated supply of non-acute care, Ireland, 2014.

Figure 1 presents the distribution of each of the non-acute services by county, detailing the variability in supply across counties and the variability in the shape of the distributions across services.

Figure 1a also shows that there are some counties with consistently low levels of per capita supply for many of the services. Table 2 provides further detail on this aspect. For each non-acute service, Table 2 presents the counties where per capita supply is at least 10% higher than the national average (white circles), counties where per capita supply is at least 10% lower than the national average (black circles), and counties where per capita supply is within 10% of the national average (grey circles). Focusing on those areas with a location quotient that is lower than 0.90, a number of counties had low levels of supply relative to the national value for nearly all of the 10 non-acute services. These counties are mainly located in the East of the country, including: Kildare, Wicklow and Meath, which are the three main commuter counties around the capital city (Dublin); Wexford, Kilkenny and Waterford in the Southeast. As an exception to this eastern trend, the western county of Clare had seven non-acute services with a location quotient lower than 0.90. There was also consistency across services in terms of the areas that have high levels of supply relative to the national value. Sligo, Leitrim, Westmeath all have seven or more non-acute services, with location quotients greater than 1.10. Sligo and Leitrim are located in the north west of the country, while Westmeath is in the midlands.

**Table 2.** Supply of non-acute healthcare services, relative to the national average per capita supply (unadjusted population) in Ireland by county, 2014.

	Non-Acute Primary and Community Care								Care for Older People		Number of Services >10 per cent below
	GP	PHN/CN	PT ^1^	OT	SLT	P&C	CO&PSY	SW	LTRC ^2^	HCH ^2^	
Kildare	⚫	⚫	⚫	⚫	⚫	⚫	⚫	⚫	◯	⚫	9
Meath	⚫	⚫	⚫	⚫	⚫	⚫	⚫	⚫	⚪	◯	8
Wexford	⚫	⚫	⚫	⚫	⚫	⚫	⚪	⚫	⚫	⚪	8
Wicklow	⚪	⚫	⚫	⚫	⚫	⚫	⚫	⚫	◯	⚫	8
Clare	⚫	⚪	⚫	⚫	⚫	◯	⚫	⚫	⚪	⚫	7
Kilkenny	⚫	⚪	⚫	⚪	⚫	⚫	⚫	⚫	◯	⚫	7
Waterford	◯	⚫	⚫	⚫	⚫	⚫	⚫	⚪	⚪	⚫	7
Offaly	⚪	⚪	⚫	⚫	⚫	◯	⚪	⚫	⚪	⚫	5
Carlow	◯	⚪	⚫	◯	⚫	⚫	⚫	⚫	⚪	⚪	5
Laois	⚫	⚪	⚫	◯	⚪	⚫	◯	◯	⚪	⚫	4
Limerick	⚪	⚫	◯	⚪	⚫	⚫	◯	⚪	⚪	⚪	3
Mayo	◯	⚪	⚪	⚪	◯	⚫	⚪	⚫	⚪	⚫	3
Tipperary North	⚪	◯	⚪	⚫	⚪	◯	⚫	⚫	◯	⚪	3
Dublin North	⚫	⚪	⚪	◯	⚪	⚫	◯	◯	⚫	◯	3
Kerry	◯	◯	◯	⚪	⚪	⚫	⚫	⚪	⚫	◯	3
Monaghan	⚫	◯	⚫	◯	⚪	◯	⚫	⚪	⚪	◯	3
Longford	⚪	◯	◯	⚪	◯	⚫	◯	⚫	◯	⚫	3
Roscommon	⚫	◯	⚪	⚪	◯	⚫	⚫	◯	◯	◯	3
Dublin South	⚪	⚪	◯	◯	⚪	⚫	⚪	◯	⚪	⚫	2
Cavan	⚪	◯	⚫	⚪	⚪	◯	⚫	⚪	◯	◯	2
Louth	⚪	⚪	⚪	◯	⚫	⚫	◯	◯	⚪	◯	2
Donegal	⚪	◯	⚫	⚪	⚪	◯	◯	◯	⚫	◯	2
Tipperary South	⚪	◯	⚪	⚪	◯	⚫	⚪	◯	⚪	⚪	1
Westmeath	⚪	◯	◯	◯	◯	◯	◯	⚪	◯	⚫	1
Leitrim	⚫	◯	◯	◯	◯	◯	◯	◯	⚪	◯	1
Sligo	◯	◯	⚪	◯	◯	◯	◯	◯	⚫	◯	1
Cork	◯	⚪	⚪	⚪	⚪	◯	◯	⚪	⚪	◯	0
Galway	◯	⚪	⚪	⚪	⚪	◯	◯	◯	◯	⚪	0

Sources:   Secondary data collated by the authors ◯    County has supply at least 10 per cent *higher* than national average ⚫   County has supply at least 10 per cent *lower* than national average ⚪   County has supply approximately equal to the national average                 1. Physiotherapy supply includes public and private supply                2. Supply of long-term residential care beds per 1,000 population aged 65+; supply of home care hours per population aged 65+

The main exception to this regional pattern is the supply of LTRC. Areas with the lowest levels of supply for most of the non-acute services had relatively high levels of supply of long-term care (e.g., Kildare, Kilkenny, and Wicklow) and vice versa (e.g., Sligo).

### Inequity in supply

Following adjustment for need, the geographic distribution of each non-acute service remained unequal and overall, there was very little change in the distributions following adjustment.

**Figure 2.**
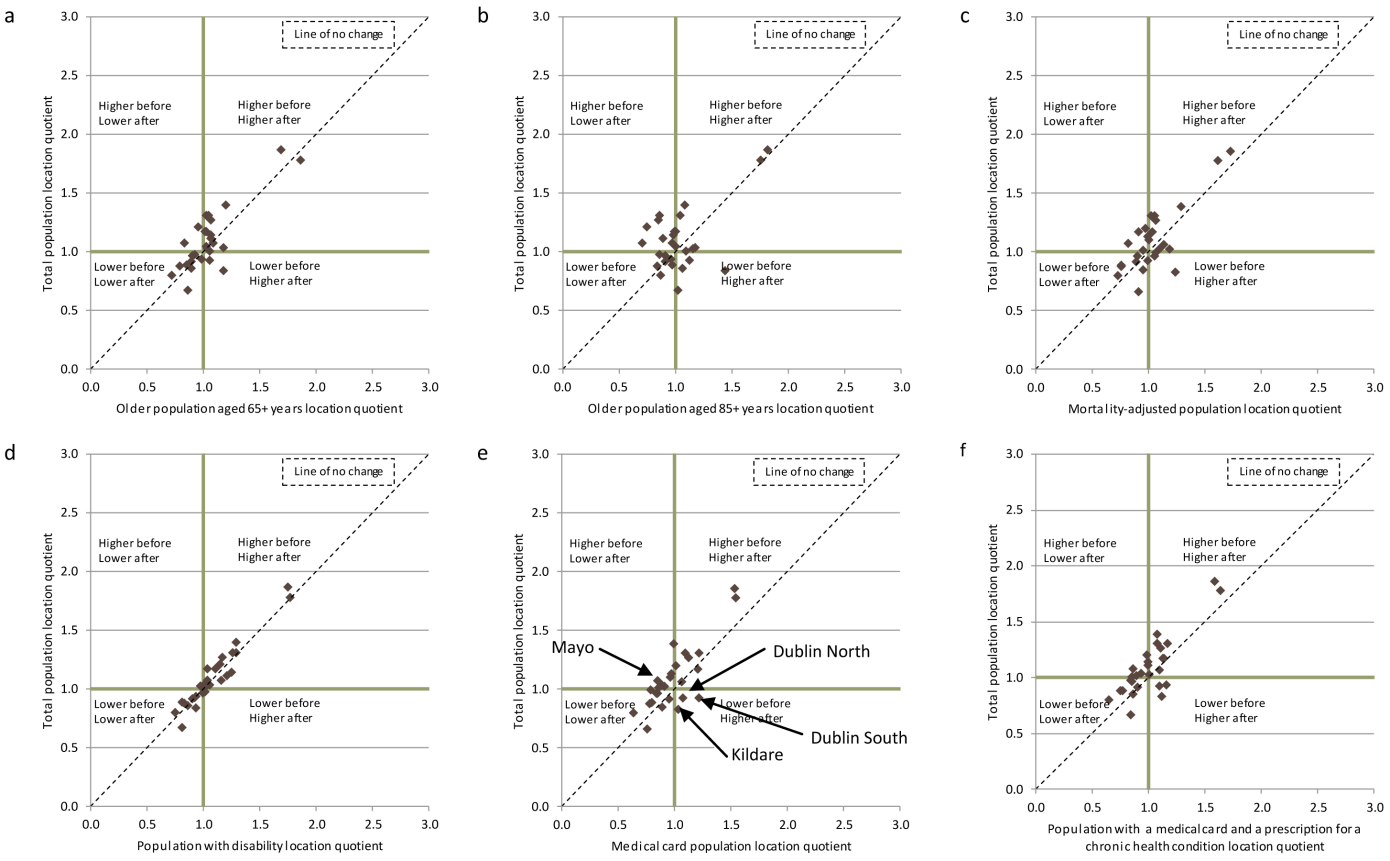
Scatter plot of location quotients of the number of WTE community nurses per 10,000 total population, and per 10,000 needs-adjusted populations by county, Ireland 2014.

The scatterplots in Figure 2 summarise how the geographic distribution of the supply of publicly employed CNs changes after adjustment for each need indicator. The graphs plot each county’s location quotient before adjustment (y axis), and after adjustment for the need indicator (e.g., MCs) (x axis). For example, a data point in the upper left quadrant indicates a county where per capita supply is higher than the national value based on the unadjusted population, and lower than the national value based on the needs-adjusted population. If needs-adjustment does not change a county’s location quotient (i.e., ratio of county to national supply), then the data point will lie on the diagonal ‘line of no change’.

For the supply of publicly employed CNs, the location quotients for the majority of counties lie close to the line of no change for each need indicator. There are exceptions to this pattern. For example, in Figure 2e, Kildare, Dublin North and Dublin South are located inside the bottom right quadrant where the location quotient was below the national average prior to adjustment for MCs (used as a proxy for healthcare need as well as socioeconomic status) (0.83, 0.93, 0.93) and higher than the national average after adjusting for the MC population (1.04, 1.08, 1.21). Conversely, Mayo is located in the top left quadrant where the location quotient fell from 1.07 prior to adjustment to 0.85 after adjustment, for the MC population. However, the analysis adopts a conservative approach of assuming that location quotients that fall within 10% of the national value are similar to the national average. Thus, these exceptions, while notable, are not considered very significant changes given that the values did not all fall outside the 10% interval.

**Figure 3.**
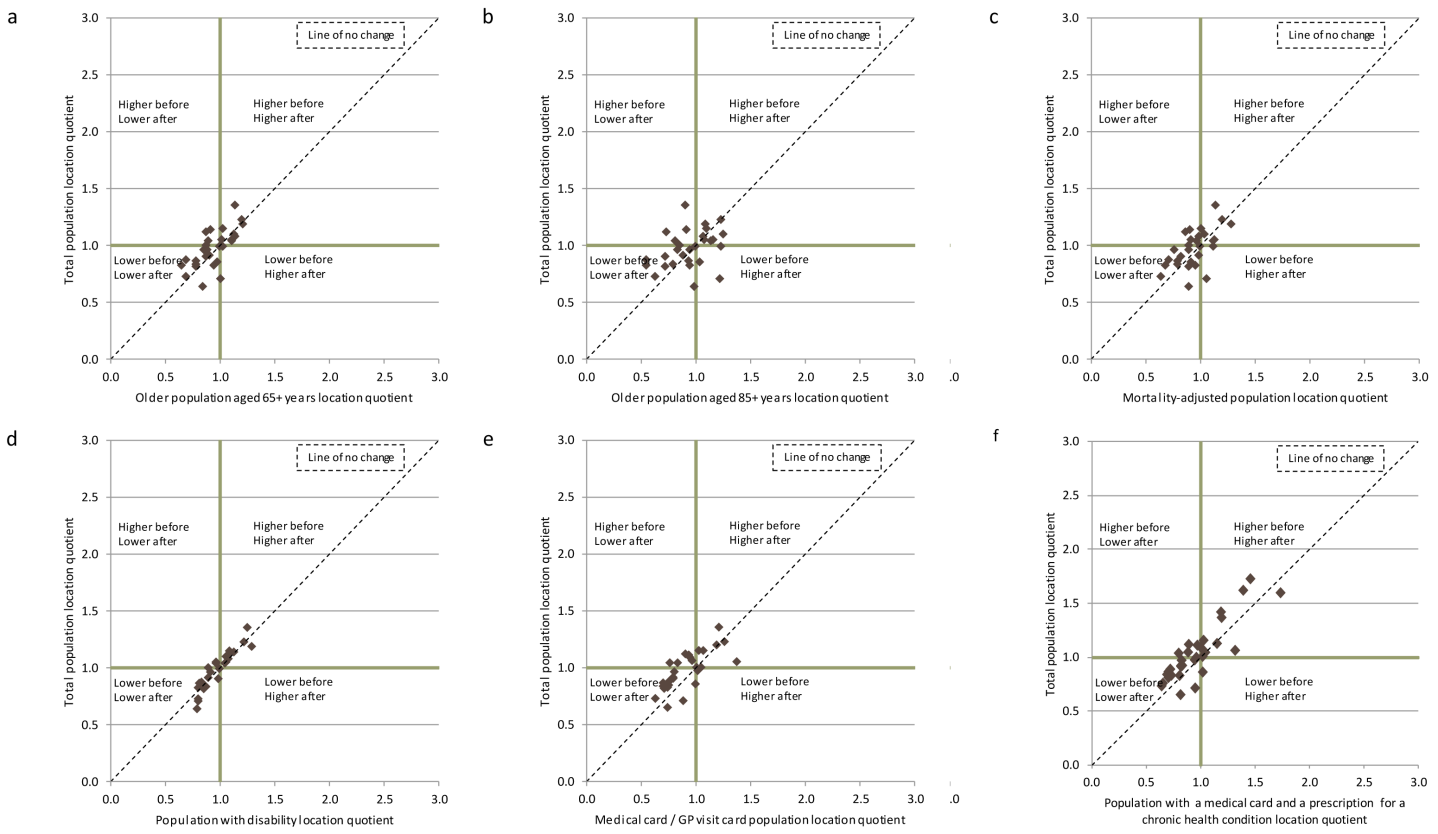
Scatter plot of location quotients of the estimated number of WTE GPs per 10,000 total population, and per 10,000 needs-adjusted populations by county, Ireland 2014.

**Figure 4.**
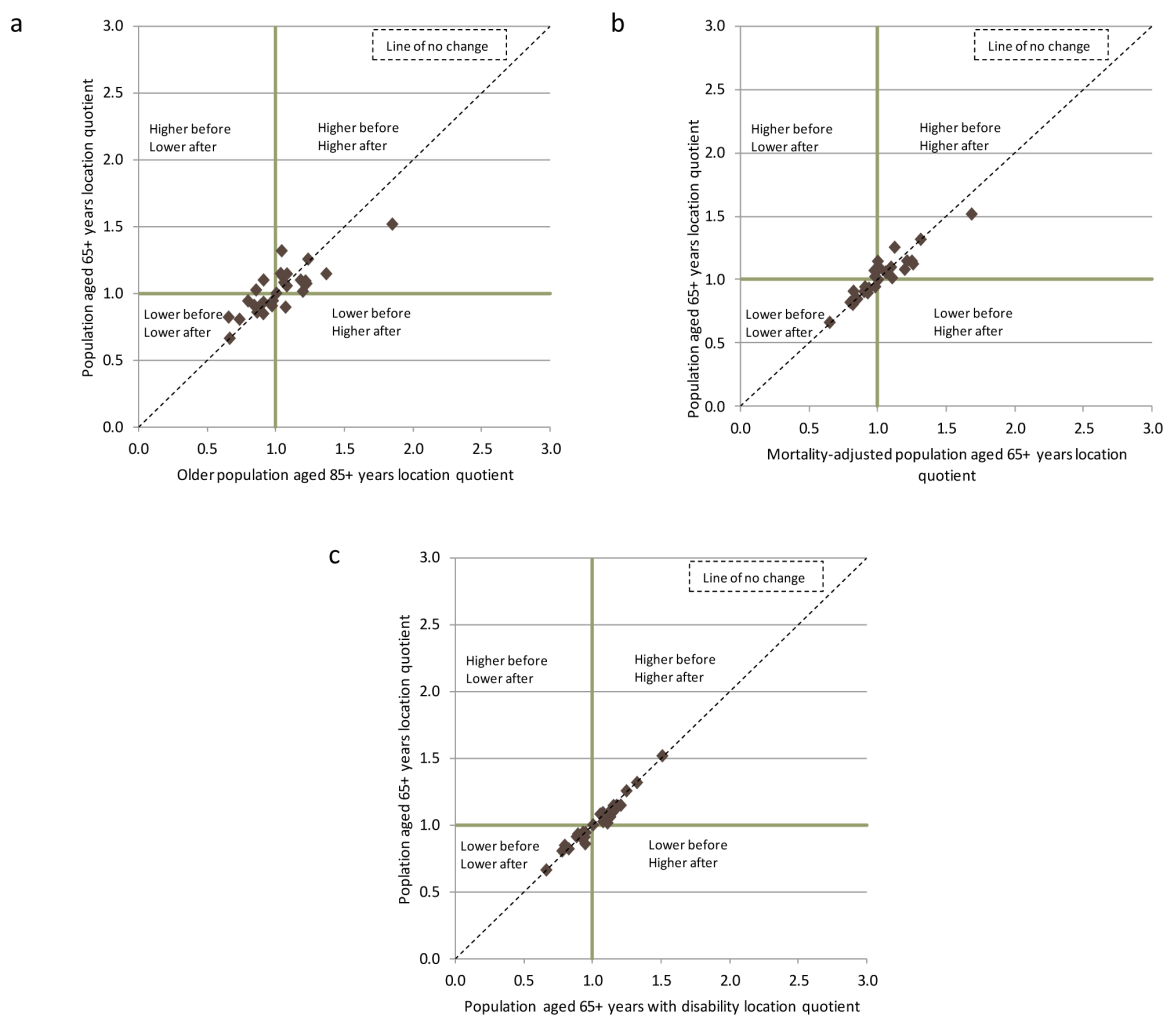
Scatter plot of location quotients of the number of long-term residential care beds per 1,000 population aged 65+ years, and per 1,000 needs-adjusted populations by geographic area, Ireland 2014.

When comparing across the different adjustment factors, for each type of supply, controlling for the population aged 85+ is most likely to influence rankings, illustrated in Figure 2 for community nursing (and Figure 3 and Figure 4 for GPs and LTRC). For each type of supply, the majority of the counties did not move from their respective quadrants following adjustment for the need indicators (although they may move within that quadrant). Kildare, Mayo and Roscommon were the most frequent exceptions to these patterns, mainly after adjustment for age 85+, but the changes in quadrants were only significant in a limited number of instances. These findings indicate that the analysis is picking up on genuine geographic inequities in supply that cannot be explained by needs.

## Discussion

### Core findings

This paper provides the most comprehensive picture of the geographic distribution of non-acute care in Ireland to date. Despite Ireland being a small country with a mainly centralised health system, the findings show considerable geographic inequalities in the supply of non-acute care across Irish counties. There are some counties that have low levels of supply of not only one, but several types of non-acute care, which include publicly employed, but also some privately provided services (GPs and private PTs). Similarly, some areas have high levels of relative supply of multiple types of non-acute care. The findings remain largely unchanged after adjusting for different needs indicators, suggesting that the unequal patterns of supply are also inequitable and have been determined by non-need factors.

With the exception of LTRC, the supply of non-acute services in Ireland is consistently lower in the east of the country compared with other regions. This pattern holds after needs adjustment. In contrast, there is more regional variation in the areas that have consistently high levels of supply (before and after needs adjustment).

While explaining the reasons behind the observed inequalities is beyond the scope of this paper, population change provides some explanation for the observed ‘eastern’ effect. There have been sizeable changes in the distribution of the population by county over the past 30 years. In particular, there have been increases in population in Dublin and the greater Dublin commuter counties. Kildare and Meath populations increased, as a proportion of national population, by more than 40% from 1986 to 2016 ^49^. Wicklow (12%) and Wexford (9%), in the east, also experienced large increases in population. The relatively low levels of per capita supply in these counties could be explained, at least in part, by supply distribution not keeping up with changes in population distribution. Over the same time period, some of the largest declines in population, as a proportion of national population, occurred in Sligo and Leitrim (12–13% reduction) which may explain why per capita non-acute supply is consistently higher than in other counties.

However, there are exceptions to these general patterns, with other factors also likely to be at play, and because of the persistence of historical budgeting it is important to keep in mind influences from former administrative structures.

The difference in the geographic distribution of LTRC compared with the other non-acute services is assumed to be influenced by location decision factors. More than 75% of LTRC beds in Ireland are now privately provided ^50, 51^. This may make equal distribution of these services more difficult because supply is likely to be influenced by market factors such as land prices, profit margins, and availability of staff.

### Limitations

Equity in access to healthcare is a core objective in many healthcare systems including Ireland ^4^, and it is acknowledged that supply is just one element within the broad concept of ‘access’ ^11^. The focus on supply in this paper is a feature of the data available for non-acute services in Ireland. In contrast, data collection on acute care and lifestyle survey data ^52, 53^ has been more extensive. For some non-acute services such as GPs and LTRC, where data can be geocoded, more detailed analysis of spatial accessibility has been undertaken ^24, 25, 27, 41^. The need for timely access to information to support an integrated healthcare system has been underlined in international policy ^3^. The emphasis in this paper has been on collating and cleaning data from disparate sources, using simple provider-to-population ratios (raw and needs-adjusted) for a broad spectrum of services to give a comprehensive picture of regional inequity in non-acute supply in Ireland.

Supply in each county is assessed against national average supply but the latter is not equivalent to adequate supply. Analysis was focused on those counties that are more than 10% different from the national average, ensuring a conservative approach is adopted to measuring variations across counties. To judge whether or not a given level of supply is adequate would require assessment against a set of agreed criteria. International supply benchmarks are challenging to determine, given the varying roles and definitions of care providers in different countries. Alternative benchmarks could include clinical standards outlining optimal care levels. For example, Wren *et al.* ^54^ examined the quality of rehabilitation care for stroke survivors in Ireland against international best practice protocols. Optimal supply may be higher than the national average for some of the non-acute services examined here, given clear evidence of unmet need and long waiting times for public community therapy services and home care ^30^.

Data gaps remain, including on the supply of private health professionals (e.g., SLTs, P&Cs, HCHs) and GP practice nurses. New data sources are coming on stream, which could be used for future analysis (e.g., the multi-profession regulator (CORU) responsible for the registration of health and social care professionals ^55^). There may also be local variations in non-acute service delivery that are not apparent in WTE data. For example, in some areas the delivery of selected non-acute services is contracted out to private providers, or there can be differing degrees of role substitution/overlapping (e.g., overlapping roles of community nurses, PTs and OTs) across counties.

Finally, the needs adjustment methods do not control for collinearity across needs indicators. However, the MC can be considered a multi-dimensional need indicator because, as well as being a key enabling factor for receiving publicly funded healthcare, the MC is also frequently considered a control for socio-economic status (since eligibility is largely based on income means-testing) ^56, 57^ and a proxy for healthcare need, given the well-established link between lower socio-economic status and poorer health ^58– 60^.

## Conclusions

### Lessons for Ireland and other countries

Formal resource allocation models for healthcare have been in use for decades ^9^, yet in Ireland, historical budgeting in non-acute care persists. The analysis in this paper shows that in the context of important population changes and the influence of non-need factors (e.g., hangovers from previous administrative structures), persistence of historical budgeting can lead to considerable geographic inequities in non-acute healthcare supply. In the Irish case, there are notable regional patterns to the observed inequities, with supply in the east consistently lower relative to the rest of the country. While such inequities can remain under the radar under normal conditions of demand (particularly in the absence of systematic analysis of supply), they come into sharp relief in the context of a crisis such as the COVID-19 pandemic where non-acute supply plays a crucial role in ensuring that acute services can be preserved for treating COVID-19 and other patients with acute care needs. A key objective in Ireland’s current health reform programme, Sláintecare ^4^, is the development of a community-costing programme for non-acute care. This, together with building capacity in non-acute care in the areas where it is most needed, is now more important than ever.

For Ireland, keeping pace with population change is critical. Future migration within and into Ireland, as well as population growth patterns, may exacerbate the regional disparities in non-acute supply, especially because projected population increases are for those areas with already relatively low levels of non-acute supply ^61^.

The Irish case also has lessons for policymakers seeking to find the appropriate balance between local autonomy and national oversight in healthcare planning. With the persistence of historical budgeting, there remains an institutional legacy with regard to the regional-based Health Boards that were in place prior to the HSE. In addition to the factors already discussed, some of the geographic differences in supply may be a consequence of the historical regional Health Board structures. Local autonomy for service planning led to divergences in non-acute supply, with some of these divergences persisting and underpinning the inequities in supply across Ireland. As part of Sláintecare, the proposed establishment of RHAs means that aspects of localised decision-making will remain. This should facilitate better matching of supply with local patient demand, but it is important that there is national oversight to ensure that data infrastructure, data analysis and resource allocation mechanisms are systematic and consistent across regional structures.

## Data availability statement

### Underlying data

This paper is based on the analysis of secondary datasets, and no other data were collected as part of this research. Details on those datasets and their host institutions are outlined below:

- Population: 2014 population estimates developed at the Economic and Social Research Institute ^37, 38^
- Supply of GPs: registers hosted and managed by the Irish College of General Practitioners and Irish Medical Directory ^24, 41^
- Supply of publicly employed Public Health Nurses, community Registered General Nurses, physiotherapists, occupational therapists, speech and language therapists, podiatrists & chiropodists, counsellors & psychologists, and social workers: Health Service Personnel Census (HSPC). Data extract provided to the authors by the HSE ^46^
  Supply of private physiotherapists: register of members of the Irish Society of Chartered Physiotherapists (ISCP). Data extract provided to the authors by the ISCP ( https://www.iscp.ie/)
- Supply of care for older people: Data on the number of publicly financed home care hours provided to the authors by the HSE. Data on the supply of LTRC beds were provided to the authors by the HSE based on combined datasets maintained by the Health Information and Quality Authority (HIQA) (responsible for regulation in long-term care), and the Department of Health (DOH). For access contact Social Care Division, Department of Health ( https://www.gov.ie/en/organisation-information/7137c8-social-care-division/).
- Mortality and disability rates: Central Statistics Office (CSO), https://www.cso.ie/en/census/census2011reports/census2011profile8ourbillofhealth-healthdisabilityandcarersinireland/; https://www.cso.ie/en/releasesandpublications/ep/p-vsys/vitalstatisticsyearlysummary2014/
- Medical cards and GP Visit card numbers: Primary Care Reimbursement Service (PCRS), https://www.sspcrs.ie/portal/annual-reporting
- Chronic illness levels: data analysed at the Department of Pharmacology and Therapeutics, Trinity Centre for Health Sciences in St. James's Hospital, Dublin and provided to the authors for this study. Access to the analysed and anonymised tables that were provided to the authors were restricted to the purposes of this study.
