# Supplementary material for: Geographic inequalities in non-acute healthcare supply: evidence from Ireland
Source: HRB Open Res. 2021 Oct 4;4:111. [Version 1] doi: 10.12688/hrbopenres.13412.1 (PMC8927742; doi:10.12688/hrbopenres.13412.1)
Supplement: Peer review history

# Peer review: Reviewer response for version 1

**DOI:** 10.21956/hrbopenres.14612.r31576

**Published:** 2022-03-16

**Authors:** Karyn Morrissey

**Affiliations:** 1 Sustainability Division, Department of Technology, Management and Economics, Technical University of Denmark, Lyngby, Denmark

**Major comments:**

- I think it would be good to explain the regional imbalance in Ireland somewhere. The economic dominance of the greater Dublin area. The aging population in the NW and West of Ireland. Higher rates of poverty outside of Dublin, etc.
- My main concern comment is that looking at needs in terms of age only is too limited. Data on current reported long term illness is available at the small area level for Ireland. This should be used as a needs indicator as well as the age profile. 
- Similarly, given the role of socioeconomic status and deprivation on health outcomes it is completely remiss not to include an indicator of need based on some sort of proxy for deprivation. The Irish index of deprivation would be ideal.
- As need should be the only driver of actual supply, I'm not sure why the authors present the non adjusted needs analysis. Or if they feel it is necessary, why the scatterplots with the line of no change? What does this dimension actually add? I really think this scatterplots are unnecessary and actually distracting. Instead I would spend more time articulating need based on a wider range of variables as I note.

**Minor comments:**

- Make it clear in the abstract that needs are based on age profile of units of analysis.
- Provide a table displaying the 10 services of interest and high level data - number per county/
- Provide an overview of each county in terms of the Irish Index of Multiple deprivation, not just age and population. 
- I really think you need to add a map of the 26 admin countries for international readers.
- Where was data on addresses obtained? Is there finally a database or was it via web scraping?

Is the work clearly and accurately presented and does it cite the current literature?

Yes

If applicable, is the statistical analysis and its interpretation appropriate?

Yes

Are all the source data underlying the results available to ensure full reproducibility?

Yes

Is the study design appropriate and is the work technically sound?

Yes

Are the conclusions drawn adequately supported by the results?

Yes

Are sufficient details of methods and analysis provided to allow replication by others?

Yes

Reviewer Expertise:

Population health, access to health services, Non communicable Diseases

I confirm that I have read this submission and believe that I have an appropriate level of expertise to confirm that it is of an acceptable scientific standard, however I have significant reservations, as outlined above.

# Peer review: Reviewer response for version 1

**DOI:** 10.21956/hrbopenres.14612.r31578

**Published:** 2022-03-11

**Authors:** Andres Garchitorena

**Affiliations:** 1 UMR 224 MIVEGEC, Univ. Montpellier-CNRS-IRD, Montpellier, France

The article by Smith and collaborators presents an interesting analysis of geographic inequalities in the distribution of primary care (termed “non-acute healthcare” because it goes beyond GPs and community health) in Ireland. The article is clearly presented, the methods are well described and appropriate, and the results are interesting, given the lack of literature on the subject. The manuscript has been thoroughly reviewed by a previous researcher and it seems that some of the recommendations have been taken into account in the current version. I will not go into too much detail or repeat things that were already said, because I feel the article is strong and does not need major revisions. I only have a few minor comments that could help clarify further some remaining elements and improve the presentation of the paper. These are summarized below:

**Methods:**

- Since ESRI can be interpreted as the company that owns ArcGIS, especially when talking about GIS data, I would use the full name of the institute to avoid confusion. You only use it twice anyway, so it is not worth the risk.
- The adjustment for morbidity is not explained in enough detail that one can know exactly how it was done. If I understand it, the authors use data on people with a MC card going to one hospital in the capital to get the % of people with chronic conditions, and from there, extrapolate to other areas based on the % of people with MC cards. Is that right? If so, couldn’t this approach overestimate or in any case bias their estimates as it comes from a hospital setting (not the general population) and from the capital city (may not be representative of rural areas)? It is worth adding some more details about the methods and rationale for this choice.
- Even though the use of Gini coefficients is common in the literature on inequalities, it would be useful for the reader to briefly describe how to interpret values of these coefficients.
- A small formatting issue: “Methods” is used both as the headline of the section and the headline of a subsection within the “Methods” section, which is confusing.

**Results:**

- In figures 2, 3 and 4, the range in the axes is too large. The authors could improve visualisation and interpretation if the ranges were reduced to 0.5-1.5 (or up to 2 for some). In addition, to facilitate interpretation of results and give more information, dots in the graph could be color-coded or numbered, so that patterns seen in Fig1 and Table 1, can also have an equivalent in terms of this adjusted information in figures 2, 3 and 4.
- As the authors explain, some of the geographic inequalities are “spatial”: consistently, areas in the east of Ireland have lower supply of services, as well as commuters around the capital. It would be really nice to see these results in a map of Ireland, as non-Irish readers do not necessarily know the location of those different counties and cannot otherwise make those conclusions just by looking at the names of counties.

**Discussion/Conclusion:**

- A main hypothesis for the inequalities observed, which is highlighted from introduction to conclusion, is that historical budgeting has not kept up with changes in population growth. Even if this is not the main goal of the paper, it could be relevant to include a very basic analysis showing this relationship (e.g. a plot of population growth by county against supply of non-acute care in 2014).
- I expected to see some comparisons of the work presented here for Ireland with similar work done in other countries, in order to put the study in the broader context of the literature on the subject. Is Ireland unique in its approach to non-acute care? Have other studies in European countries found similar/different results as observed here? Have they approached the topic of inequalities in non-acute care similarly, or in different ways, or not at all?
- In terms of health policy, do the authors have some specific recommendations based on the results observed and the specific context of the Irish health system? I wonder particularly how governments (local or national) can help reduce existing gaps given that a large part of the medical workforce seems either private or at least not subject to allocation by centralized planning.

Is the work clearly and accurately presented and does it cite the current literature?

Yes

If applicable, is the statistical analysis and its interpretation appropriate?

Yes

Are all the source data underlying the results available to ensure full reproducibility?

Yes

Is the study design appropriate and is the work technically sound?

Yes

Are the conclusions drawn adequately supported by the results?

Yes

Are sufficient details of methods and analysis provided to allow replication by others?

Yes

Reviewer Expertise:

Global health, health systems strengthening

I confirm that I have read this submission and believe that I have an appropriate level of expertise to confirm that it is of an acceptable scientific standard.

# Peer review: Reviewer response for version 1

**DOI:** 10.21956/hrbopenres.14612.r31331

**Published:** 2022-02-09

**Authors:** Ronan Foley

**Affiliations:** 1 Department of Geography, National University of Ireland, Maynooth, Maynooth, Ireland; 2 Department of Geography, Maynooth University, Maynooth, Ireland

**GENERAL:**

 This is a very interesting paper that collates and visualizes very effectively an assemblage of data from across non-acute healthcare supply and draws from it some important insights on spatial variations from within that data. The context of the work is clear, especially in relation to how it might inform the development of reform within the current system. While there is some good literature cited around spatial accessibility, many of the GIS-driven spatial analytical approaches of that work are not used with the authors preferring a more spatial economics approach; but perhaps it would be good to make more explicit in the text a loss of nuance in using tools such as location quotients; even though the latter are visualized in interesting if complex form. It is also the case that that wider literature cited was working with better geo-spatial data in more constrained service settings so completely understand that choice. The data collection seems pretty sound within the constraints of what the authors have to work with, and the analysis of the spatial variation, again very acceptable given similar methodological constraints.

 I have a number of broad observations and reflections and some specific questions about the geographical decisions made on the development of the data and some wider conclusions but I will detail these below. Some of the comments might seem to argue for a reworking of the data analysis but I am not minded to ask the authors to do this, rather maybe incorporate a bit more meaningful geographical thinking into the paper as a whole. In addition I think there is a real potential, especially given the authorship of this paper, to push a wider agenda around more creative thinking on geographical data scales, collection techniques and coding. This is, as the authors note, beyond the scope of this paper, yet the paper makes a subtle but powerful case for a deeper examination of same in the future and why not include that as a statement in the final section at least.

**SPECIFIC COMMENTS:**

 A first comment is that while it is sensible to focus on a single year, 2014, its choice as the year of study is not clearly justified in the paper. A year that might align with a census, say 2016, might make more sense, but if the data collected does not align, then that’s fine but maybe clarify a little. Indeed the need to coalesce the analysis around data that is 7-8 years old is in itself a rather telling comment on the available of meaningful spatial data across the primary/community sector.

- **Introduction:**
  - Introduction is very efficient and presents the investigation clearly and within good health care politics and data contexts. While the paper does code up the data sets as public and private later on, I wonder whether some sort of sense of where Slaintecare is coming from in being a response to the current complex braided tiers (depending on service) of service supply, might help for a wider readership beyond Ireland, who might be less familiar with the complexities of our public/private model; something that is perhaps even more deeply embedded in the PCCC side as it is on the hospitals side.
  - On the data, I think there is a very valuable point to make, and you do so in an almost over-subtle way, on the lack of availability of private service supply data. This remains a real structural barrier to meaningful integrated planning and that affects both availability and analysis in this type of modelling.
  - I like the mention of the impact of historical budgeting, which might even be broadened out to the sort of long-run historical development of primary and community services more  generally. That slow geographical development of services over time also shapes something you pick up on later in terms of spatial inequalities, namely the low ranking of new commuter geographies on terms of service supply compared to places with more established histories and this is a very nice finding that links the two together.
- **Background:** As noted in the general comments, there’s a nice efficient summary here of the different ways in which accessibility and service supply emerge in both spatial and aspatial ways and nice to see some of the GIS specific literature mentioned here as well. Central to that literature is work around travel time, catchments and indeed cross-boundary mobilities in service accessibility and utilization. The paper does not really use any of that literature, focusing instead on slightly older measures but I do agree that the data constraints you are working with make these more contemporary geospatial methods hard to apply and you note they also do not fit exactly to your aims and good to see. In terms of the paper’s listed aims, the first two make sense, the third one mostly so but not quite sure what exactly the last one is saying?
- **Institutional context: **Nice efficient summary here of the complex history of health service boundaries and also the core structures that shape access good to see but given paper is built around services in 2014, might be useful to clarify which boundaries were extant then, and what is indeed the current state of play in 2022, even if that is still pretty up in the air. Just a clarity around how the dates line up.
- **Geographic setting:**
  - This is one area I have a little difficulty with – the use of what one might argue are a mix of out-of-date and also randomly allocated counties feels odd to me. Why not use Local Authorities, especially in Dublin, where the old county had not been a meaningful entity for several decades now? The creation of a random South and North Dublin also splits Dublin City, one of the core new local authorities, in two parts. Equally prior to 2014, most of the other Irish cities were separate local authorities to their associated counties – by loosely amalgamating them, you arguably may lose some important urban/rural nuance beyond the Great Dublin area. But perhaps this is another rationale for your choice of 2014 as the core date for analysis? I can see you needed to use counties to probably reflect in part how data is still, fairly lazily recorded in the system but would like to see you make a stronger case for the specific geography you use. I do appreciate that later in the document you do also note that LHO boundaries are not coterminous with the old counties, and indeed that some sort of spatial retro fitting was needed. This is presented as something slightly trivial, but given the complexity of LHOs in Dublin and Cork and the double-counties in some cases, this was I suspect quite a task for the Health Intelligence Unit to develop. So I do get you end up with a slightly unsatisfactory compromise between service and admin boundaries and I think I would like you to just say that, given how the data forces you to work.
  - The listing of ‘ten’ supply variables is slightly confusingly listed in the text, though much clearer in Table 1 on the next page. It might be a little pedantic, but could you maybe number/code them in the text as they are listed in turn in page 4 as well.
- **Methods: **The use of GINI coefficients for a general service variation measure is very interesting, and while challenging to interpret at times, the location quotients are used in interesting ways. I also like the weighting variables and I think I can see the sense in showing the raw and weighted versions in the graphs on pages 8, 9, and 10. I wonder why you don’t just present the weighted version as a stand alone visualisation but I do get that you want to provide some nuance to what can otherwise be a fairly blunt instrument in its raw form.
- **Results: **
  - I really like the graphics on pages 6 and 7 and think these are really clever visualisations. While the geographer in me would like to see at least one map in the paper, if only to provide a non-Irish (and indeed many Irish) readers with the actual geographical boundaries and what they look like, I can see from a data perspective, Figure 1 and Table 1 work really well. There’s a lot of complex data crammed in here and if anything I might have liked a bit more discussion on these findings, but very clever visualisation work here and nice to get that sense of an accumulation/assemblage of spatial inequalities across services you get from both. The analysis of this data seems to me something I would like to see develop a little – what’s nice about the data is that it picks up the specific under-supply in the commuter counties of the east, but think this is also a pattern evident in many of the other bigger cities as well, especially Cork and Galway and that’s got a little lost in the amalgamated counties mix.  More importantly, in that first paragraph on page 8 you actually talk about geography and why it might matter or explain/shape the observed results.
  - With the different analysis of the LQs, it very nice to see that on Figure 2e at least the counties are given names and are not mere dots on a graph. I wonder if the same might be applied to some of the other outliers in other sub-charts, though I do get that you suggest all the data is within an acceptable level of variation. Also just in terms of the variables used in weighting, I don’t think you mention deprivation much in the paper at all. I completely understand the impossibility of applying any sort of ‘average’ dep score from smaller units to a county scale, but given the literature on the impacts of COVID-19 on these areas, it might be worth a mention in passing, if only to explain why it was not used because of geo-spatial modelling difficulties. This would also bring in a sense of how post-COVID inequalities may become further embedded in terms of the services you discuss.
- **Discussion:** I do think there is quite a difference between the different measures you use, though I do appreciate that both long-term residential and home care are listed as ‘services’. From that point of view, its good to see you note that the geographical variations with these two measures are differently aligned than most of the others. Other readers might argue for the exclusion of these two which might make the other results hold together but I think its precisely the invisibility of these strands of non-acute care that argue for their inclusion and well done for building them in.
- **Limitations: **You do mention some data as being geocoded, but I think it would be a lost opportunity for the paper in looking forward, to make no mention of Eircodes. Given these are point based, a better integration of these into data records would make for much more open amalgamation of data into newer service geographies, such as the 96 new Community Health Networks. This in turn speaks to a wider potential for more nuanced analysis at a sub-county level in future work. While its clear from your work that even pulling together disparate data sets at a county scale is hard enough, the geospatial modelling potential of aggregating records collected at Eircode level to any new geographical reporting unit is actually one of the genuine values of the Eircode design. Some mention of the potential and need for new spatial coding and modelling to finer geographical scales seems an implicit finding from the paper; why not make it more explicit to get people who develop data in the non-acute sector to consider this more seriously

Is the work clearly and accurately presented and does it cite the current literature?

Yes

If applicable, is the statistical analysis and its interpretation appropriate?

Yes

Are all the source data underlying the results available to ensure full reproducibility?

Yes

Is the study design appropriate and is the work technically sound?

Yes

Are the conclusions drawn adequately supported by the results?

Yes

Are sufficient details of methods and analysis provided to allow replication by others?

Yes

Reviewer Expertise:

Medical/Health Geography; GIS and Health

I confirm that I have read this submission and believe that I have an appropriate level of expertise to confirm that it is of an acceptable scientific standard, however I have significant reservations, as outlined above.
